# Refining Biologically Inconsistent Segmentation Masks with Masked Autoencoders

**DOI:** 10.1109/CVPRW63382.2024.00684

**Published:** 2024-06-17

**Authors:** Alexander Sauer, Yuan Tian, Joerg Bewersdorf, Jens Rittscher

**Affiliations:** 1 Department of Engineering Science, University of Oxford; 2 Department of Cell Biology, Yale School of Medicine

## Abstract

Microscopy images often feature regions of low signal-to-noise ratio (SNR) which leads to a considerable amount of ambiguity in the correct corresponding segmentation. This ambiguity can introduce inconsistencies in the segmentation mask which violate known biological constraints. In this work, we present a methodology which identifies areas of low SNR and refines the segmentation masks such that they are consistent with biological structures. Low SNR regions with uncertain segmentation are detected using model ensembling and selectively restored by a masked autoencoder (MAE) which leverages information about well-imaged surrounding areas. The prior knowledge of biologically consistent segmentation masks is directly learned from the data.

We validate our approach in the context of analysing intracellular structures, specifically by refining segmentation masks of mitochondria in expansion microscopy images with a global staining.

## 1 Introduction

Modern microscopes enable precise visualization and analysis of cellular structures and biological processes. However, microscopy images are often affected by uneven signal quality. This can be due to various reasons like the biological sample itself and its preparation, the staining process, inadequate exposure times or photon-induced bleaching. Such degradation in image quality not only compromises the visual examination of biological specimens but also impedes subsequent analysis steps. For example, the resulting regions of low SNR pose a challenge for computer generated segmentation masks as they offer substantially more room for interpretation and this ambiguity results in the lack of a well defined corresponding segmentation ground truth.

**Figure 1:**
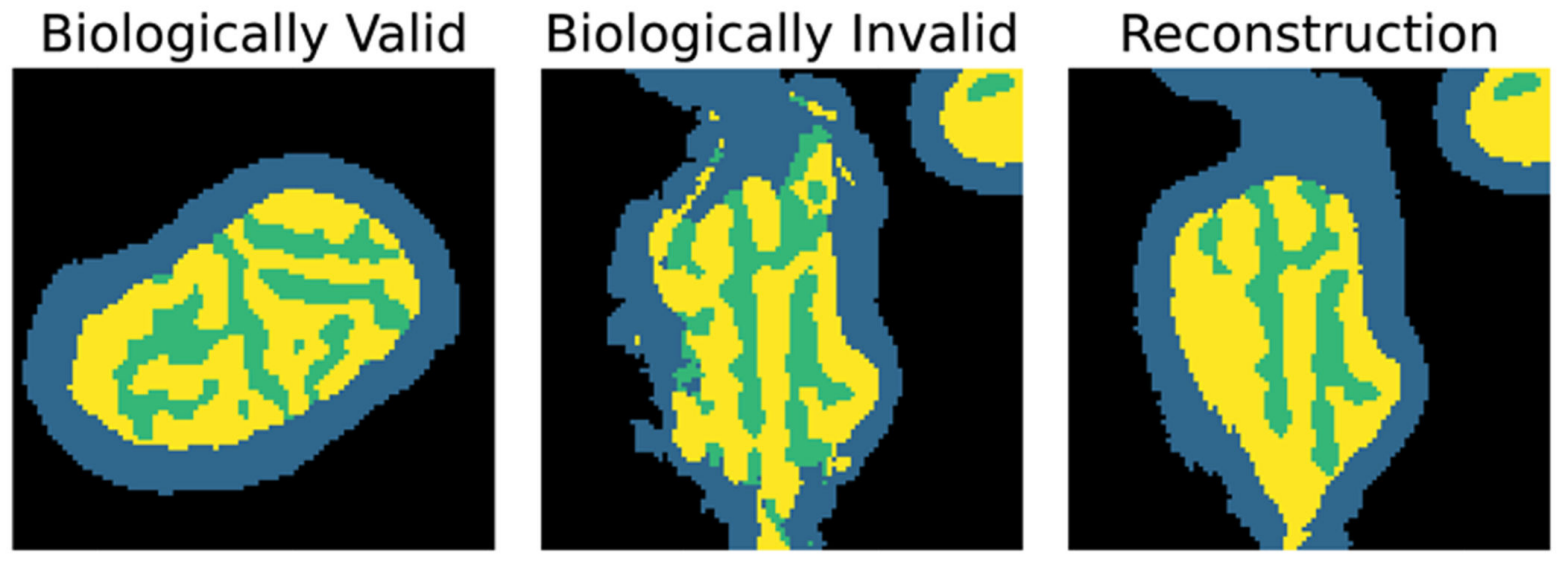
Comparison of a biologically valid segmentation mask, a biologically invalid segmentation masks and our proposed reconstruction Left: Example showing a segmentation mask which is biologically plausible showing *cristae* invaginations (green) surrounded by *matrix* (yellow), the inner membrane being surrounded by *intermembrane space* (blue) and overall smooth boundaries. Middle: A segmentation mask that violates the biological constraints with separated *cristae* areas and fragmented boundaries. Right: The same example adhering to biological constraints again after applying our MAE refinement.

Nevertheless, the inherent self-similarity of biological structures often provide constraints to perform effective signal amplification and refinement. In this paper, we use the concrete example of mitochondria imaging to propose a new post-processing framework for resolving highly ambiguous regions of poor signal quality to enhance the phenotypical analysis of cellular organelles. As depicted in Figure 1, it is known that mitochondria are formed by a double membrane structure [19] and these membranes typically form smooth structures without any fragmentation. Moreover, the inner membrane forms invaginations, called *cristae*, which therefore should mostly be surrounded by area of the inner membrane, called *matrix*, and cannot appear independently.

The typical approach for image segmentation is to predict a probability distribution over the possible classes for each pixel and output the class with the highest likelihood. If the underlying image offers a lot of ambiguity, however, the segmentation model can produce segmentation masks which are not coherent with the biological prior knowledge as dependencies among labels are only indirectly accounted for through the shared context.

**Figure 2:**
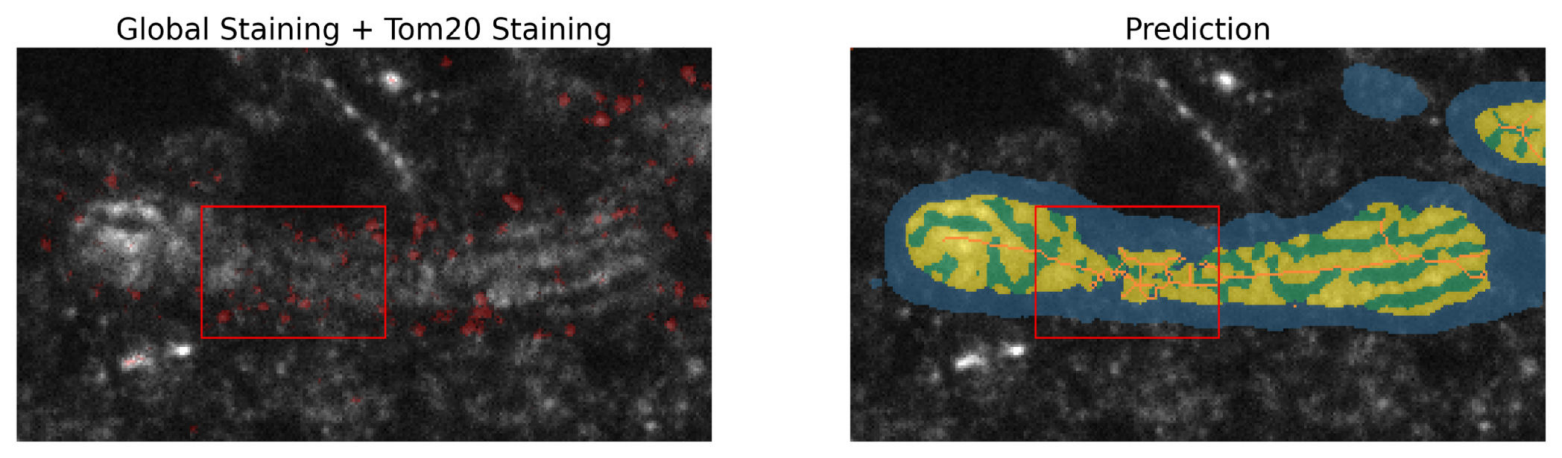
Poor segmentation as a consequence of low SNR The red rectangle highlights an area in which the global staining [18] is unclear and shows how this leads a segmentation mask that is biologically inconsistent. This introduces artifacts in subsequent analysis steps like the skeletonisation [16] which is shown in orange. The Tom20 staining in red (which is known to bind to the outer membrane of the mitochondria) shows that the highlighted area is still part of the inner membrane and not outside or on the boundary. This Tom20 staining is not used in any subsequent analysis.

These segmentation mistakes are problematic as they can introduce artifacts in subsequent analysis steps. The example in Figure 2 shows how the resulting skeletonisation of the inner structure of the mitochondria shows several loops and branches which would bias any resulting length measurement without additional post-processing steps.

Addressing this challenge can be divided into two sub-problems: how can we identify areas of possibly poor staining quality and subsequently, what is the appropriate strategy to deal with them? Sticking with the example of mitochondria, one naive approach would be to exclude all mitochondria which are somewhere affected by some poor imaging. However, this introduces a significant bias into any subsequent analysis: As longer mitochondria occupy more area, they are more likely to be affected by some imaging issue and therefore more likely to be removed.

Previous attempts have been made to impose shape priors into segmentation models [2]. However, formulating these priors is challenging as they have to be fine-tuned manually and the resulting methods suffer from long run times unsuitable for the large volumes of data produced by modern microscopes. On the other hand, recent advances in machine learning suggest that learning such priors directly from the data offers a superior approach.

When human experts are confronted with a small area of high ambiguity, they annotate the sample to be consistent with the well-imaged surrounding area and their knowledge of the underlying biological structure. This ability has been gained by having seen a large number of coherent samples and is made possible by a large degree of local self-similarity between the mitochondria samples.

Our method aims to imitate the process followed by a biologist and we demonstrate how it improves the segmentation quality of mitochondria imaged with a global ’pan’-staining using expansion microscopy [4, 18]. The ’pan’-staining binds to multiple proteins and generates signal throughout the entire specimen.

We first show that the disagreement between an ensemble of models provides a good approximation for the underlying image quality. Once areas of low SNR are identified, we train a masked auto-encoder (MAE) to reconstruct biologically plausible segmentation masks from a corrupted segmentation masks. For inference, we specifically mask the area of high ambiguity and use the trained MAE to reconstruct the segmentation output consistently with the surrounding area to form biologically coherent predictions. We show that our method produces more sound segmentation masks than competing post-processing methods like conditional random fields (CRF) and leads to fewer artifacts in the subsequent analysis.

## 2 Related work

### 2.1 Uncertainty detection

Common methods to quantify prediction uncertainty include Bayesian neural networks (BNN), Monte Carlo drop-out methods and deep ensembles [1, 8]. While BNN [10] offer a theoretically elegant solution to capture the uncertainty in the model weights, they suffer from high computational complexity. Monte Carlo drop-out methods [7] approximate the posterior predictive distribution by multiple forward passes of the same input through the network while omitting random subsets of neurons, but might be poorly calibrated and sensitive to hyper-parameter choices [3].

Deep ensembles [14] are conceptually simple as they only require retraining the same model with different initialisations and the prediction uncertainty is computed by investigating the difference in the individual predictions.

### 2.2 Shape Constraints

Popular approaches to include shape priors in segmentation models are conditional random fields (CRF) and active contour models (ACM) [2]. CRFs are typically used as a post processing step and build a neighbourhood graph that allows to incorporate information about adjacent pixels into the prediction. However, the approach is limited by high computational cost for large neighbourhoods except when additional constraints are imposed on the neighbourhood influence [13].

ACMs on the other hand are based on the idea of iteratively transforming an initial outline to minimise some energy function. This energy function again needs to be fine-tuned to the application at hand and the resulting models have a limited adaptability to datasets showing a large variety of shapes and object sizes as typically encountered with the inner structure of mitochondria.

Another approach attempts to capture the shape in a lower dimensional space through an autoencoder. Larrazabal et al. [15] pass the initial segmentation mask through an autoencoder to smooth out low frequency information to regularise the final shape. However, compared to our method, their approach works globally without focusing on ambiguous areas and requires handcrafting a deterioration function to train the autoencoder.

## 3 Method

In order to formalise our method, let us denote the globally stained image by *X* ∈ ℝ^*H*×*W*^ where *H, W* denote the height and width of the image. Moreover, we denote the set of possible segmentation labels by 𝒞 and the associated segmentation mask by *Y* ∈ 𝒞^*H*×*W*^.

The standard practice for image segmentation in the deep learning framework is to train a network *f*_*θ*_ parameterised by *θ* to predict a probability distribution ${\text{ℙ}}_{f_{\theta }}[Y_{i}|X]$ over 𝒞 for each pixel *i* given the input *X*. The final prediction is subsequently chosen as the label which maximises this probability ${\hat{y}}_{i}=\text{arg }{\text{max}}_{c\in C}{\text{ ℙ}}_{f_{\theta }}\left[Y_{i}=c|X\right]$.

This approach is based on the assumption that conditional on the input *X*, the pixel labels are independent and we can factorise the overall probability over individual pixels ℙ[*Y* |*X*] = Π_*i*_ ℙ[*Y*_*i*_|*X*].

However, let us consider pixel *i* in more detail and rewrite the global probability accurately as

(1) $$\text{ℙ}[Y|X]=\text{ℙ}\left[Y_{i}|X,Y_{-i}\right]\text{ℙ}\left[Y_{-i}|X\right]$$

where *Y*_−*i*_ denotes all remaining pixels. If pixel *i* is poorly stained and shows a low SNR, *X* provides little guidance for the associated segmentation label of this pixel. Therefore, in this work we explore the assumption that it is therefore better predicted by its surrounding pixels, i.e. ℙ[*Y*_*i*_|*X, Y*_−*i*_] might be better approximated by ℙ[*Y*_*i*_|*Y*_−*i*_] than ℙ[*Y*_*i*_|*X*] for poorly stained pixels. When we denote the area of unclear staining by *U* and the remaining, reliable pixels by *R*, we therefore propose to predict the final segmentation according to the factorization

(2) $$\text{ℙ}\left[Y|X\right]=\text{ℙ}\left[Y_{U}|Y_{R}\right]\cdot \prod_{i\in R}\text{ℙ}\left[Y_{i}|X\right]$$

in which we follow the conditional independence assumption only for reliable pixels in *R*.

**Figure 3:**
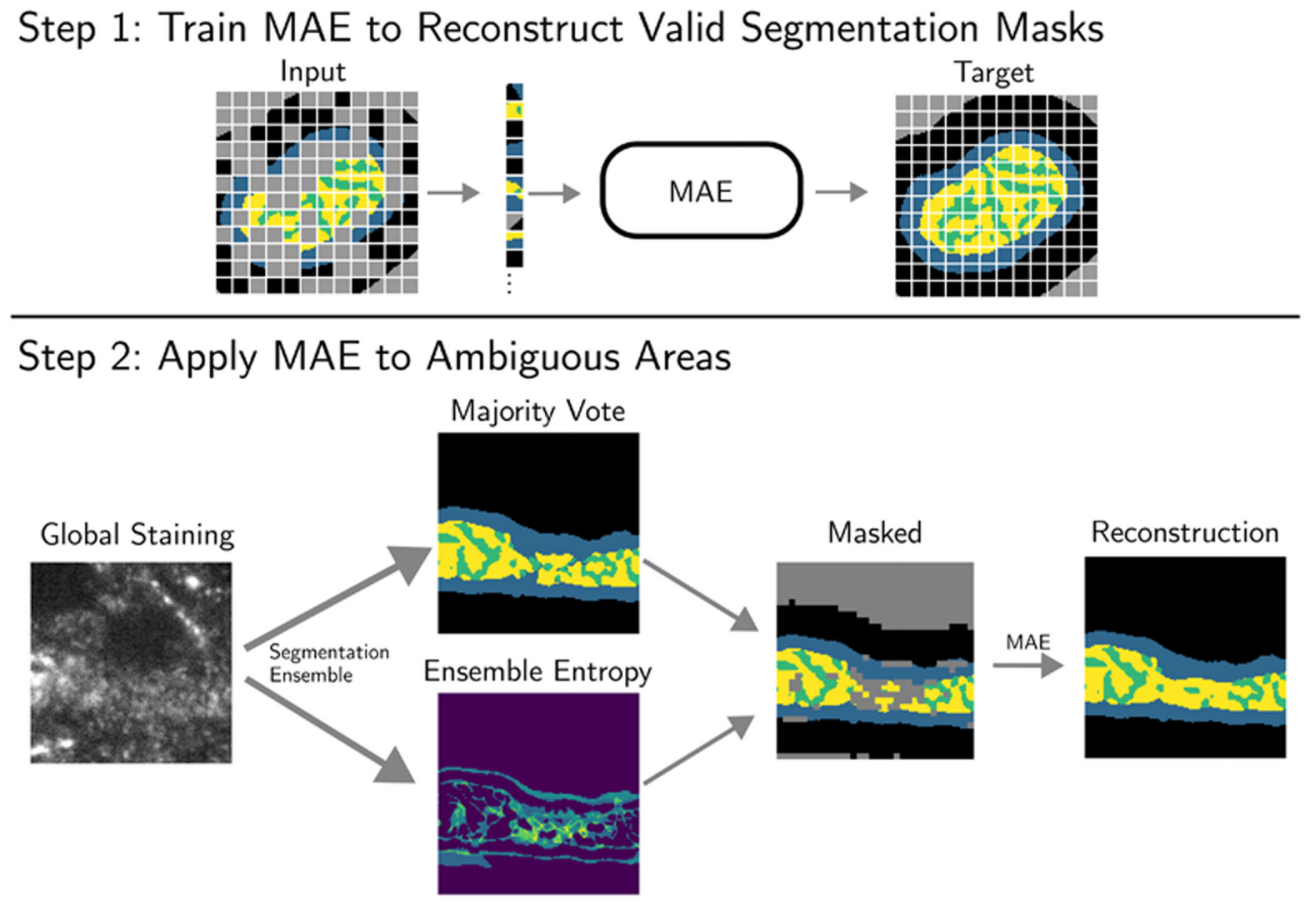
In the first step we train a Masked-Autoencoder (MAE) to construct biologically coherent segmentation masks from partial segmentations while ignoring random patches (greyed out). In the second step, we apply an ensemble of models to the global staining to compute the majority vote and ensemble entropy. Subsequently, we remove the patches with a high ensemble entropy and reconstruct them using the MAE. Background patches far from the segmentation mask are ignored as well. The shown example patch corresponds to the red rectangle from Figure 2.

This approach leaves us with two tasks: Firstly, finding the pixels with potentially ambiguous staining and therefore possibly inconsistent segmentations and secondly, learning the unconditional distribution *P*(*Y*_*U*_|*Y*_*R*_) for applying a prior of consistent annotations to this area. Our proposed pipeline is summarised in Figure 3.

### 3.1 Identification of Low SNR

We define an area of unclear staining as a set of pixels *U* ⊂ {1, …, *H* · *W*} in which *X* does not carry enough information by itself to produce an unambiguous segmentation mask *Y*_*U*_. This implies that in a well calibrated model, we would expect the predicted probability ℙ[*Y*_*U*_|*X*] to be fairly uniform and therefore have a high entropy. However, deep learning based learning are known to be overly confident [11].

We therefore use the prediction entropy of an ensemble of models trained on different subsets of the annotated data as deep ensembles have been shown to be able of identifying uncertain predictions more accurately [1, 3, 5]. We expect such capability to be even stronger in our setting as we can exploit that the annotated training data also includes ambiguous areas. This allows to create a diverse set of models within the ensemble as individual models settle on different associations between ambiguous areas and the segmentation labels during their training process.

Moreover, this method aligns well with our approach of a post-processing step as it does not require to modify the original segmentation algorithm.

To capture the disagreement among models in the ensemble we compute the average prediction ${\hat{p}}_{i}^{c}$ over the models for class *c* and pixel *i*. Subsequently, we compute the Shannon entropy $H_{i}=-{\text{Σ}}_{c=1}^{C}\;{\hat{p}}_{i}^{c}\text{log}\left({\hat{p}}_{i}^{c}\right)$ which is normalised into [0, 1] as our indicator of the associated uncertainty.

### 3.2 Segmentation Prior

Once the ambiguous areas in the overall image with potential inconsistent segmentations are identified, we predict these pixels based on its surroundings following ℙ(*Y*_*i*_|*Y*_−*i*_) instead of ℙ(*Y*_*i*_|*X*).

Our aim is to learn an approximation to ℙ(*Y*_*i*_|*Y*_−*i*_) = *∫* ℙ(*Y*_*i*_|*x, Y*_−*i*_)ℙ_*V*_ (*x*)*dx* as a prior for what biologically valid segmentation masks look like by integrating ℙ(*Y*_*i*_| *x, Y*_−*i*_) over the distribution of biologically plausible image patches ℙ_*V*_ (*x*). The intuition behind this approach is that the segmentation masks of the mitochondria show a high degree of local self-similarity. Therefore, given a large pool of mitochondria, it is possible to learn a model that is able to reconstruct small missing areas coherently from its surroundings.

#### Inference

While MAEs were originally designed as a self-supervised pre-learning technique, we use it as a generative model and apply it directly to the segmentation mask of an initial segmentation model. Specially, after the MAE is trained, we use the predictions of the initial segmentation model to generate a first segmentation mask and identify areas of possible unclear staining based on the ensemble entropy. We then divide the initial segmentation of size *W* × *H* into *L* smaller patches of size *w* × *h* and filter out patches with a mean uncertainty above some hyperparameter *τ*_*thres*_. The remaining patches are forwarded through the MAE to reconstruct the uncertain patches and filled in to produce the final segmentation as shown in Figure 3.

This approach is motivated by the observation that MAEs are able to act as very efficient generative models [20]. Moreover, while implementing this method with a convolutional neural network needs careful consideration in order to avoid information leakage [17] from the masked areas, the architectures of a vision transformers (ViT) [6] and masked autoencoders (MAE) [12] align very closely with our requirement of ignoring specific areas in the input and allow for a straight forward implementation.

#### Training

The strategy to train the MAE for ℙ(*Y*_*i*_|*Y*_−*i*_) is to first randomly mask out areas and reconstruct the clearly stained areas from the remaining pixels. Given a masking ratio *ρ*, a random subset of *ρ* · *L* of all patches are removed and only the randomly selected patches are augmented by a positional encoding and then forwarded through an encoder model *h*. Moreover, we want to encourage the model to be able to work with a variety of masking ratios as the size of the entropy mask in the inference step varies and therefore sample *ρ* ~ 𝒰[0.1, 0.9] uniformly.

The encoder is a ViT which first transforms the patches into a latent feature $z_{0}\in {\text{ℝ}}^{d_{z}}$ which subsequently is passed through a series of *m* multiheaded self-attention, MLP and normalisation blocks to produce the final latent representation *z*_*m*_. These features are subsequently concatenated with a learnable mask token for every disregarded patch, the positional encoder is added and forwarded through a decoder build on the same architecture as the encoder.

The output of the decoder is passed through a final linear layer that transforms the output into prediction probabilities [0, 1]^|𝒞|^. The MAE is then trained to match the original segmentation as closely as possible in terms of the cross-entropy loss. In order to ensure that the model only learns coherent segmentations, the loss function only includes pixels *i* which have an entropy value *H*_*i*_ smaller than some threshold *τ*_*train*_ while the labels of uncertain pixels are ignored. Additionally, we ignore pixels which are too far from any mitochondrion in order to prevent the loss from being dominated by background pixels.

Comparing our method to graphical models like CRFs, the encoder based on the self-attention blocks can be interpreted as constructing a global graph between all present patches. The random masking scheme therefore corresponds to sampling random subgraphs and learning how to complete them. However, in contrast to CRFs in which the interactions between different vertices needs to be handcrafted, the MAE allows to learn those dependencies automatically from the data in order to be able to reconstruct the missing patches.

## 4 Experiments

### 4.1 Data

We apply our method to pan-Expansion Microscopy (ExM) images [18]. The idea behind expansion microscopy is to physically expand the sample to enable nano-scale resolution with commonly available microscopes [4]. Compared to other competing super-resolutions methods this technique does not require specialised hardware and therefore promises to make super-resolution imaging more accessible [9]. Pan-ExM is a variant of Expansion Microscopy that retains proteins through an iterative 20-fold expansion process and then labels all proteins with a global ‘pan’-staining. The combination of the high optical resolution enabled by the large expansion factor and the contrast that reveals protein densities as a whole enables the visualization of cellular ultrastructure similar to Electron Microscopy.

We aim to segment the double membrane structure of the mitochondria. In our segmentation figures, the *intermembrane space* between the outer and inner membrane is depicted in blue and presents itself as a dark halo around the mitochondria in the global staining. Moreover, the inner membrane forms invaginations to increase its surface area which are called *cristae* and shown in green in our figures. Finally, the space enclosed by the inner membrane is called *matrix*, shown in yellow in our figures and can be recognised by its higher intensity in the global staining.

For our experiments we use twelve image stacks of size 2048 × 2048 pixel with a z-stack depth between 74 and 234. Each stack shows parts of one HeLa cell and contains on average 150 mitochondria. While the vast majority of the mitochondria are clearly stained, this work focuses on a sub-sample of mitochondria which are partly effected by some ambiguous staining. We therefore use all of the available data for training but focus our evaluation on a subset of 100 patches of size 128 × 128 which visually show clear anomalies and compare them to a set of 100 control patches which show coherent segmentation masks.

The initial segmentation and entropy masks are provided by an ensemble of 3D U-Nets trained on a small amount of manually annotated data.

### 4.2 Implementation Details

The overall images we consider are of size 128 × 128 and the sub-patches have size of 4 × 4 pixels. The encoder of the MAE has a latent dimension of 128 and a depth of 14. Following the original MAE implementation [12], we use a lighter decoder with a latent dimension of 64 and a depth of 7. Both models use a twice as many latent dimensions for the hidden layer of the MLP.

For the identification of reliable areas in the training we use a threshold of *τ*_*train*_ = 0.1 while the threshold for the inference step is set at *τ*_*thres*_ = 0.4. An implementation of our method is available at https://github.com/AlexSauer/BioMaeRef.

### 4.3 Results

#### Identifying Uncertain Area

**Figure 4:**
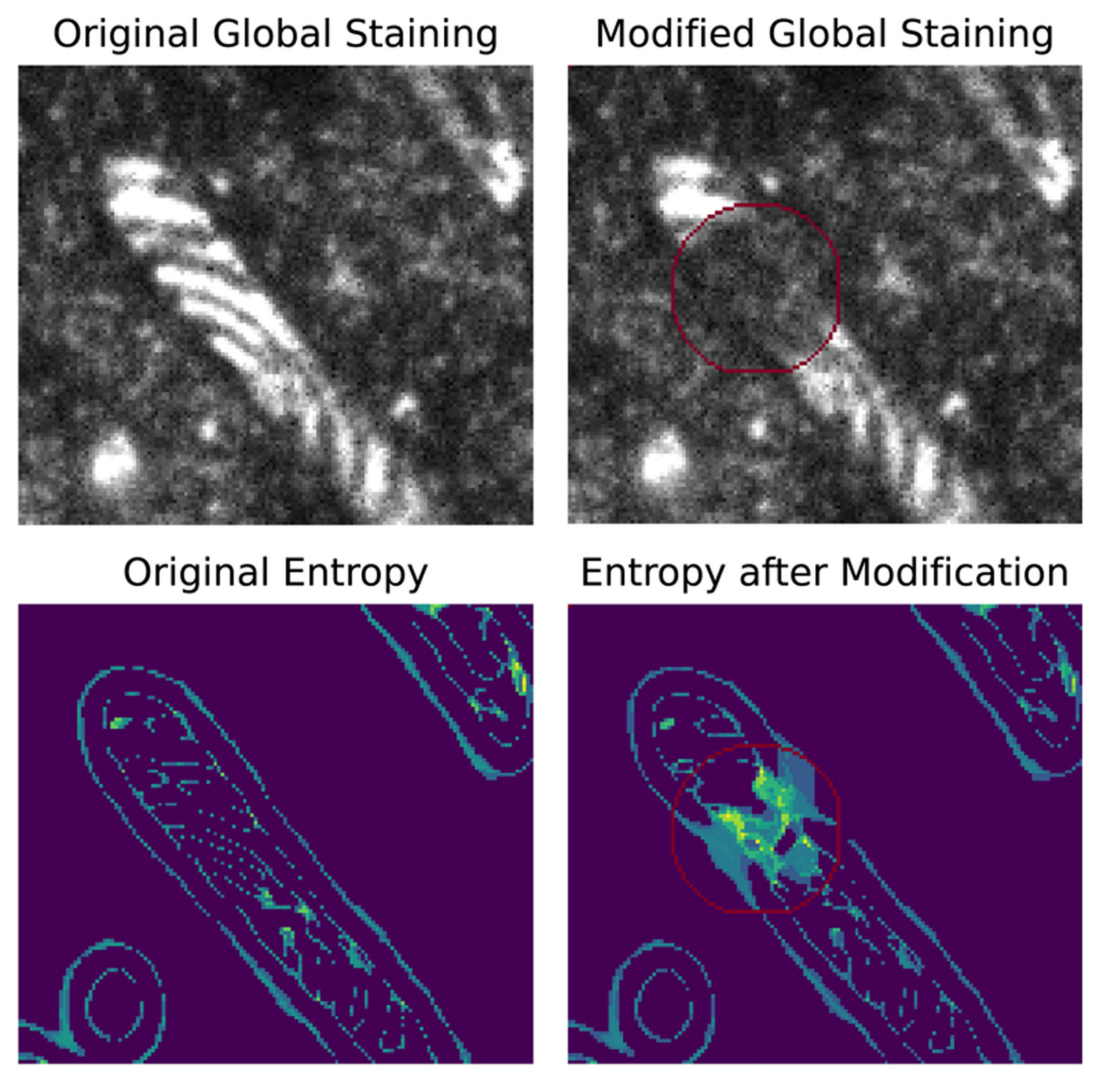
Simulation of unclear staining The top row shows a global staining patch before and after we degrade the imaging quality in the area highlighted by the red circle. The bottom row shows the corresponding ensemble entropy with brighter colors corresponding to higher values. The quality degradation leads to a substantial increase in the predicted uncertainty in the modified area.

In order to test our assumption that poorly imaged areas in the global staining correspond to an increase in the ensemble entropy, we conduct experiments in which we artificially degrade the imagining quality for some areas and measure the change in the prediction consistency as shown by Figure 4.

Specifically, we create a mask along the skeleton of the mitochondrion which is indicated by a red circle in Figure 4. We then take a patch consisting purely of background and compute the linear interpolation between the original mitochondrion, some Gaussian noise and the background to simulate a situation with unclear global staining. This interpolation is weighted by the distance map of the mask in order to ensure a smooth transition between the original image and the added background. The induced change can be seen in the top row of Figure 4.

As it can be seen in the lower row of Figure 4, we see a substantial increase in the entropy in the corresponding area. To quantify the effect in more detail, we repeat the above procedure for 100 mitochondria and find an increase of 160% in the entropy associated with these areas and we see that areas that were in the lowest 10% of entropy original are within the top 60% of entropy after the modification. Additionally, Figure 6 shows how areas of poor signal in the global staining are related to areas of inconsistent initial segmentations and are reliably picked up by the entropy mask.

#### Reconstruction of plausible areas

While the previous experiment suggests that our approach has a good performance of correctly identifying areas of unclear staining and therefore possible implausible segmentations, it might also accidentally create false positives and flag areas which have been segmented consistently. We therefore test the reconstruction quality of our method for plausible segmentation masks by randomly dropping patches from the control group of plausible mitochondria masks and compute the resulting reconstruction accuracy. Table 1 shows that even under high masking ratios we are able to reconstruct accurate segmentation mask due to the high self-similarities of the biologically valid structures.

#### Evaluation

**Figure 5:**
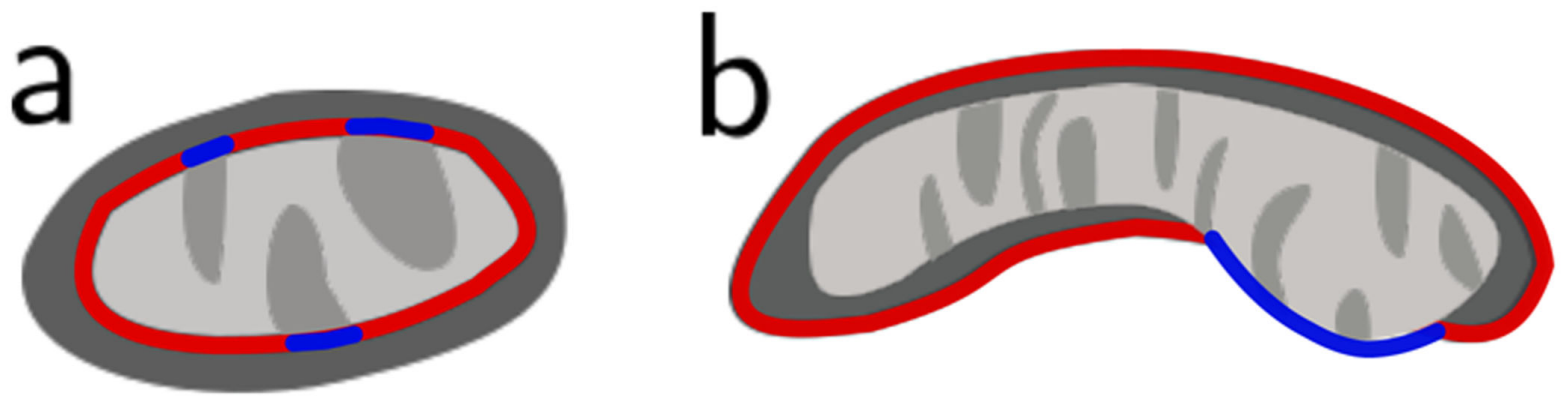
Validity metrics a: The ’Cristae Boundary Ratio’ $\frac{\;\text{blue}\;}{\;\text{blue}\;+\;\text{red}\;}$ is the proportion of *cristae* (blue) in the boundary between the inner structure (*cristae* + *matrix*) and the *intermembrane space* (blue + red). b: The ’Inner Membrane Background Ratio’ $\frac{\;\text{blue}\;}{\;\text{blue}\;+\;\text{red}\;}$ refers to the proportion of the boundary between the segmentation mask and the background (blue + red) which does not consist of *intermembrane space* (blue)).

In order to evaluate our entire post-processing pipeline shown in Figure 3, we focus on several metrics that approximate whether the segmentation mask adheres to the biological constraints: We previously discussed that a biologically valid segmentation mask should show most of the *cristae* area surrounded by *matrix* area and should include some *intermembrane space* between the inner structure and the background. To evaluate the plausibility of the *cristae*, we therefore compute the length of the boundary that the *cristae* forms with the *intermembrane space*. To account for mitochondria of different sizes we compute the ratio of this value to the length of the overall boundary that the inner structure forms with the *intermembrane space* and call this metric **cristae boundary ratio** (Figure 5a). This metric is higher if the segmentation exhibits independent areas of *cristae*. Additionally, we compute the length of the boundary between any inner structure and the background and compute to ratio to the length of the overall boundary length between the mitochondria mask and the background. We call this metric **inner membrane background ratio** and it captures areas of missing *intermembrane space* (Figure 5b).

Moreover, in order to capture the smoothness of the mask, we calculate the ratio between the boundary length over the area and look at the number of branches that a skeleton of the inner part of mitochondrion forms.

In order to interpret these values, we additionally compute these metrics for a control group of biologically consistent segmentation masks that serve as a baseline for our method evaluation.

#### Comparison to other methods

**Figure 6:**
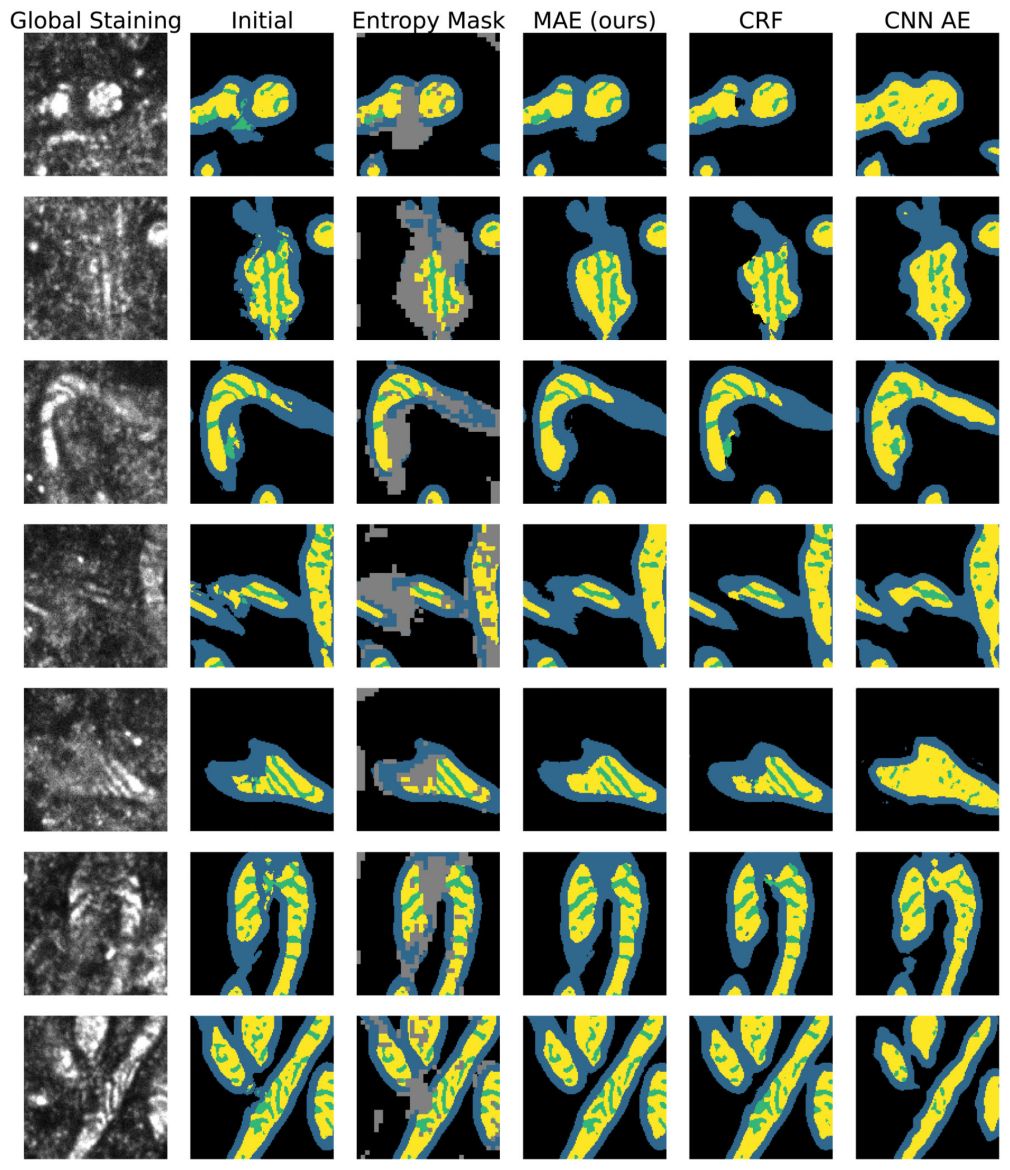
Reconstruction results Visual comparison of the initial segmentation, the patches masked based on high entropy levels, the reconstructed segmentation masks and the global staining that the initial prediction is based on. It can be seen that the MAE reconstructed masks better respect the biological constraints like no *cristae* area (green) that is detached from *matrix* area (yellow) and less fragmented boundaries.

We compare our methods against CRFs that are based on mean field approximations [13] to allow for long range dependencies and global convolutional autoencoders (CNN AE) similar to Larrazabal et al. [15]. The results are shown in Table 2 and show that the segmentation masks by our method bring the described metrics closest to the metrics of the control group of biologically valid segmentation masks. We find that CRFs are able to smooth out the segmentation boundaries but fail to reproduce biological constraints as seen by the high inner membrane background ratio which indicates that it often misses some *intermembrane space*. The CNN AE on the other hand fails to reconstruct the cristae accurately. Figure 6 shows a visual comparison of the methods.

**Test the validity of** ℙ(*Y*_*i*_|*Y*_−*i*_): In section 3 we factorised ℙ[*Y* |*X*] = ℙ[*Y*_*i*_|*X, Y*_−*i*_] ℙ[*Y*_−*i*_|*X*] and argued that for poorly stained regions ℙ[*Y*_*i*_|*Y*_−*i*_] might be a better target than ℙ[*Y*_*i*_|*X*], which is given by the initial segmentation method. In this section we compare how our estimate of ℙ[*Y*_*i*_|*Y*_−*i*_] compares to ℙ[*Y*_*i*_|*X, Y*_−*i*_]. In order to approximate ℙ[*Y*_*i*_| *X, Y*_−*i*_], we modify our method by attach an additional encoder with *X* as the input. This encoder follows a standard ViT encoder that takes the global staining as input without any masking and produces a latent representation for every patch. We then add this representation to the latent representation of the segmentation mask if the patch was present or to the mask token if it was not. Sub-sequently, the updated representation is forwarded through the decoder to produce the final segmentation mask. In this way, we allow our method to be able to access the global context *X* as well as the surrounding initial segmentation masks *Y*_−*i*_ and we follow the same training and inference procedure outlined in section 3.

Comparing the results in Table 2, in which the method is labeled MAE+X, shows that adding the additional context deteriorates the performance. We speculate that the model focuses too much on reproducing the association between the initial segmentation mask *Y*_*i*_ and the input *X* rather than capturing the interactions with the surrounding neighbourhood *Y*_−*i*_ during the training.

## 5 Discussion

In this work, we have proposed a post-processing framework which is capable of correcting biologically invalid segmentation masks. Our framework combines deep ensemble in order to identify areas of poor signal and leverages the strength of MAEs to learn repetitive patterns and selectively reconstruct the segmentation masks for these regions. Our methods has the advantages that it infers the structure of biologically plausible segmentation masks directly from the data and it can be applied as a post-processing step independently of the underlying primary segmentation algorithm. Moreover, the reconstruction based on the MAE requires a single forward pass and is therefore able to scale to large data volumes.

One challenge associated with our approach is that the use of strong priors can detach the prediction to some degree from the observed data. This implies that some valid structures could potentially be removed or that some of the segmentation masks which are corrected by our MAE are actually false positives and should rather be removed completely. However, since we cannot reliably differentiate between false and true positives, we argue that it is still beneficial to modify the false positives so that their influence on any subsequent analysis is limited.

While we demonstrated the effectiveness of our approach with the example of mitchondria segmentation and expansion microscopy, in future work we plan to apply the proposed method to other segmentation tasks in which the biological structures show large degree of degree of self-similarity and biological constraints.

## Figures and Tables

**Table 1:** Reconstruction accuracy Dice scores of the reconstruction for clearly stained regions using different ratios of randomly masked patches.

Masking ratio	0.1	0.25	0.5	0.75	0.9
Dice Score	0.97	0.97	0.95	0.92	0.86

**Table 2:** Segmentation evaluation with respect to biological constraints The ‘Cristae Boundary Ratio’ and ‘Inner Membrane Background Ratio’ are explained in Figure 5. The different methods are evaluated on a subset of 100 mitochondria with visually implausible segmentation masks. Additional a control group with plausible segmentation masks was evaluated to provide baseline values. Standard errors are reported in parenthesis.

Method	Skeleton End Points	Skeleton Cycles	Boundary to Area Ratio (%)	Cristae Boundary Ratio (%)	Inner Membrane Background Ratio (%)
Initial Segmentation	10.90 (0.60)	2.93 (0.37)	14.21 (0.59)	17.50 (1.19)	0.00 (0.00)
CNN AE	5.51 (0.30)	0.08 (0.04)	11.39 (0.22)	2.98 (0.34)	0.76 (0.28)
CRF	6.14 (0.34)	0.81 (0.15)	12.95 (0.49)	13.46 (1.06)	8.46 (1.48)
MAE+X	8.64 (0.50)	1.02 (0.16)	13.13 (0.64)	15.31 (0.87)	0.65 (0.18)
**MAE (ours)**	**5.05 (0.29)**	**0.18 (0.07)**	**11.41 (0.39)**	**8.71 (0.58)**	**0.36 (0.03)**
Control Group	3.55 (0.30)	0.26 (0.14)	12.14 (0.94)	10.09 (0.88)	0.00 (0.00)
